# A meta-analysis of hyperfractionated and accelerated radiotherapy and combined chemotherapy and radiotherapy regimens in unresected locally advanced squamous cell carcinoma of the head and neck

**DOI:** 10.1186/1471-2407-6-28

**Published:** 2006-01-31

**Authors:** W Budach, T Hehr, V Budach, C Belka, K Dietz

**Affiliations:** 1 Department of Radiation Oncology, University Hospital Düsseldorf, Germany; 2 Department of Radiation Oncology, University Hospital Tübingen, Germany; 3 Department of Radiation Oncology, Charité, Berlin, Germany; 4 Department of Medical Biometry, University Hospital Tübingen, Germany

## Abstract

**Background:**

Former meta-analyses have shown a survival benefit for the addition of chemotherapy (CHX) to radiotherapy (RT) and to some extent also for the use of hyperfractionated radiation therapy (HFRT) and accelerated radiation therapy (AFRT) in locally advanced squamous cell carcinoma (SCC) of the head and neck. However, the publication of new studies and the fact that many older studies that were included in these former meta-analyses used obsolete radiation doses, CHX schedules or study designs prompted us to carry out a new analysis using strict inclusion criteria.

**Methods:**

Randomised trials testing curatively intended RT (≥60 Gy in >4 weeks/>50 Gy in <4 weeks) on SCC of the oral cavity, oropharynx, hypopharynx, and larynx published as full paper or in abstract form between 1975 and 2003 were eligible. Trials comparing RT alone with concurrent or alternating chemoradiation (5-fluorouracil (5-FU), cisplatin, carboplatin, mitomycin C) were analyzed according to the employed radiation schedule and the used CHX regimen. Studies comparing conventionally fractionated radiotherapy (CFRT) with either HFRT or AFRT without CHX were separately examined. End point of the meta-analysis was overall survival.

**Results:**

Thirty-two trials with a total of 10 225 patients were included into the meta-analysis. An overall survival benefit of 12.0 months was observed for the addition of simultaneous CHX to either CFRT or HFRT/AFRT (p < 0.001). Separate analyses by cytostatic drug indicate a prolongation of survival of 24.0 months, 16.8 months, 6.7 months, and 4.0 months, respectively, for the simultaneous administration of 5-FU, cisplatin-based, carboplatin-based, and mitomycin C-based CHX to RT (each p < 0.01). Whereas no significant gain in overall survival was observed for AFRT in comparison to CFRT, a substantial prolongation of median survival (14.2 months, p < 0.001) was seen for HFRT compared to CFRT (both without CHX).

**Conclusion:**

RT combined with simultaneous 5-FU, cisplatin, carboplatin, and mitomycin C as single drug or combinations of 5-FU with one of the other drugs results in a large survival advantage irrespective the employed radiation schedule. If radiation therapy is used as single modality, hyperfractionation leads to a significant improvement of overall survival. Accelerated radiation therapy alone, especially when given as split course radiation schedule or extremely accelerated treatments with decreased total dose, does not increase overall survival.

## Background

The disappointing results of conventionally fractionated radiotherapy in locally advanced squamous cell cancer of the head caused investigators to test new treatment strategies. Based on retrospective clinical data and radiobiological considerations hyperfractionated and accelerated radiation regimens as well as chemoradiation regimens have been investigated in a large number of clinical trials. Hyperfractionation and acceleration of radiotherapy has been identified as potentially advantageous compared to conventionally fractionated radiotherapy in comprehensive reviews [1] and a former meta-analysis [2]. However, the existence of a real benefit has been challenged [3,4] and neither hyperfractionation nor acceleration has been widely accepted as standard of care. The availability of the results of a number of new studies prompted us to carry out a new meta-analysis.

The addition of chemotherapy to radiotherapy was analysed in the MACH-NC meta-analysis and showed a small but significant survival advantage in favour of chemotherapy (4% at 5 years), which was higher (8% at 5 years, hazard ratio (HR) 0.81) in case of simultaneous radiochemotherapy compared to sequential or adjuvant chemotherapy [5]. An update of this meta-analysis [6] including 87 trials and more than 16 000 patients confirmed the results of the earlier analysis. Although, some information in the MACH-NC meta-analysis is provided about relevant subgroups of studies, we felt that a more detailed look at the radiation dose and fractionation schedules and the employed chemotherapy regimens used in the chemoradiation trials is of interest. Furthermore, we think that neither studies using drugs that are no longer in clinical use in combination with concurrent radiotherapy in head and neck cancer, because of documented severely enhanced acute mucosal toxicity (bleomycin and methotrexate) nor studies using subcurative radiation schedules in the radiotherapy alone arm should be included into a meta-analysis, if one wants to get clinically meaningful conclusions. Therefore, our study group performed a meta-analysis based on randomised trials fulfilling strictly defined entry criteria that tested concurrent or alternating chemoradiation versus radiation therapy alone.

## Methods

### Eligibility criteria for clinical trials

Three groups of randomised trials on patients with squamous-cell carcinoma of the head and neck (oral cavity, oropharynx, hypopharynx, and larynx) without distant disease using radical radiotherapy in the control arms of the studies were eligible: 1. Studies comparing radiotherapy to radiotherapy in combination with chemotherapy. 2. Studies comparing conventionally fractionated radiotherapy (CFRT) to accelerated fractionated radiotherapy (AFRT). 3. Studies comparing conventionally fractionated radiotherapy to hyperfractionated radiotherapy (HFRT). No surgery other than neck dissection after primary treatment was allowed. Trials on nasopharyngeal carcinomas were excluded. Radical radiotherapy in the control arms of the studies was defined as the administration of at least 60 Gy total dose, if the overall treatment time was longer than 4 weeks, and the administration of at least 50 Gy, if the overall treatment time was below or equal to 4 weeks. Studies using lower total doses were excluded, because such treatment was considered palliative radiotherapy. More details on the eligibility criteria in the specific subgroups are given in the following paragraphs:

#### 1. Comparison of radiotherapy vs. radio-chemotherapy

Only trials comparing radiotherapy alone with radio-chemotherapy using simultaneous or alternating cisplatin, carboplatin, mitomycin C, and 5-fluorouracil (5-FU) as single drug or combinations of 5-FU with a platinum-derivate were eligible. Studies using bleomycin or methotrexate containing chemotherapy regimens were excluded, because these drugs have been shown to dramatically enhance radiation induced mucositis and skin reaction [7,8] and are therefore no longer in clinical use in combination with radiotherapy in most countries. Radio-chemotherapy trials were divided into three groups:

Group 1a included studies comparing CFRT alone in the control arms with CFRT in combination with simultaneous chemotherapy in the experimental arms of the studies. The difference in overall treatment time had to be ≤1%, and the difference in total radiation dose ≤1% between the control and the experimental arms of the studies, respectively.

Group 1b included studies comparing HFRT or AFRT alone in the control arms with HFRT or AFRT in combination with simultaneous chemotherapy in the experimental arms of the studies. To be considered as accelerated radiation more than 10 Gy total dose per week had to be administered on average during the radiation series. HFRT required the application of at least 2 daily fractions for at least one third of the radiation series. The difference in overall treatment time had to be ≤11%, and the difference in total radiation dose ≤11% between the control and the experimental arms of the studies, respectively.

Group 1c included studies with a substantial imbalance of the radiation regimen used in the control and the experimental arm of the studies. CFRT, HFRT or AFRT alone in the control arms were compared with similar fractionated radiation regimens, but large differences in overall treatment time and/or total radiation dose in combination with alternating or simultaneous chemotherapy in the experimental arms of the studies. Either the difference in overall treatment time had to be >11%, or the difference in total radiation dose had to be >11% between the control and the experimental arms of the studies.

#### 2. Comparison of conventionally fractionated radiotherapy vs. accelerated radiotherapy

Group 2 included studies comparing CFRT alone in the control arms with AFRT in the experimental arms of the studies. To be considered as accelerated radiation the overall treatment time had to be 10–50% shorter in the experimental arm of the study, if the total dose was decreased by no more than 5%, and had to be more than 50% shorter in the experimental arm of the study, if the total dose was decreased by more than 5%.

#### 3. Comparison of conventionally fractionated radiotherapy versus hyperfractionated radiotherapy

Group 3 included studies comparing CFRT alone in the control arms with HFRT in the experimental arms of the studies. Hyperfractionation was defined as twice daily radiation treatments with <1.25 Gy per fraction. Furthermore, the overall treatment time was not allowed to vary by more than 10% between treatment arms and the total radiation dose had to be increased by at least 5% in the hyperfractionated arms of the studies. Studies using hyperfractionated radiotherapy without dose escalation or even with lower total doses in the hyperfractionated arm of the study were excluded, because these studies did not exploit the inherent radiobiological advantage of hyperfractionation that allows for dose escalation.

### Literature search strategy

The meta-analysis aimed to include trials published as full paper or at least in published abstract form between January 1975 and December 2003 with overall survival data (for at least two time points or survival curves). Electronic databases (Medline, PubMed, and CancerLit) were searched to identify potentially eligible trials. In addition, reference lists of published reports, review articles, and relevant books were searched.

### Exclusion of specific studies

A study from Haffty et al. [9] was excluded, because results of surgically and non-surgically treated patients were not reported separately. A trial published by Poulsen et al. [10] on accelerated radiotherapy could not be included into the meta-analysis, because only disease specific survival, but not overall survival data has been reported. Only trials with at least 60 randomised patients were considered [11]. A list of further trials not fulfilling our entry criteria with the reasons for exclusion is given in the appendix (see additional file #1).

### Statistical methods

Overall survival was the end point of the meta-analysis. The meta-analysis was based on overall survival probabilities at 1, 2, 3 and, if available, at 5 years that were extracted from published papers, original patient's data (1 study [12]), or estimated from published Kaplan-Meier curves. Based on the sample sizes the absolute numbers of patients were calculated, who were expected to have died during the first year, the second year, the third year, during the time between the third and the fifth year, and who survived the last year of observation. Using maximum likelihood, a lognormal distribution was fitted to these numbers. The statistical model assumes that the control group and the treated group have the same standard deviation of the logarithms. For each study, a parameter for the mean was calculated for the control group. The common difference to the control group was estimated for all studies which are concerned with the same treatment. Using maximum likelihood methods, a 95% confidence interval for the gain in the median survival was calculated. Only the number of patients who were expected to survive the last year of observation (year 3 or 5) entered the calculations as censored variables. The authors are aware of the fact that this approach does not take into account censoring before the last year of observation, but the paucity of information provided in the publications left no other choice. The present method does not affect the estimates of median survival. Only the width of the confidence intervals is underestimated. The goodness of fit of the lognormal model with the observations was assessed with the chi-square test (see additional file #2). The reason to choose the gain in median overall survival as end point of the meta-analysis rather than hazard ratios is a consequence of the observation that the hazard ratio decreased significantly over time in some of the included studies. This time dependent changes of the hazard ratios in some studies did not allow for a reliable estimate of the overall hazard ratio for all studies, whereas the gain in overall survival could be reliably estimated. Using the lognormal survival distribution it is possible to transform the gain in median survival into a gain of the survival probability at two years after the beginning of therapy together with the 95% confidence intervals. For each comparison a chi-square test for heterogeneity was performed.

## Results

Thirty-two trials with a total of 10 225 patients were identified and included into the meta-analysis. Group 1a, 1b, 1c, 2 and 3 consisted of 10, 6, 3, 9, and 4 trials including 2197, 1301, 502, 4702, and 1523 patients, respectively. The exact distribution of T- and N-stages was not available for all but for most trials. TMN stages were taken from the publications without reclassification according to a more recent TMN-classification. Looking at the median values of the available data on stage distribution, 26% (range 0% to 83%) had stage III and 69% (range 0–96%) had stage IV M0 disease, 14% (range 0% to 47%) suffered from N3-lymph node involvement, indicating that the vast majority of patients treated in the trials of interest had locally advanced head and neck cancer. As expected, predominantly male patients (median percentage 85%, range 74% to 94%) had been included into the studies.

**Figure 1:**
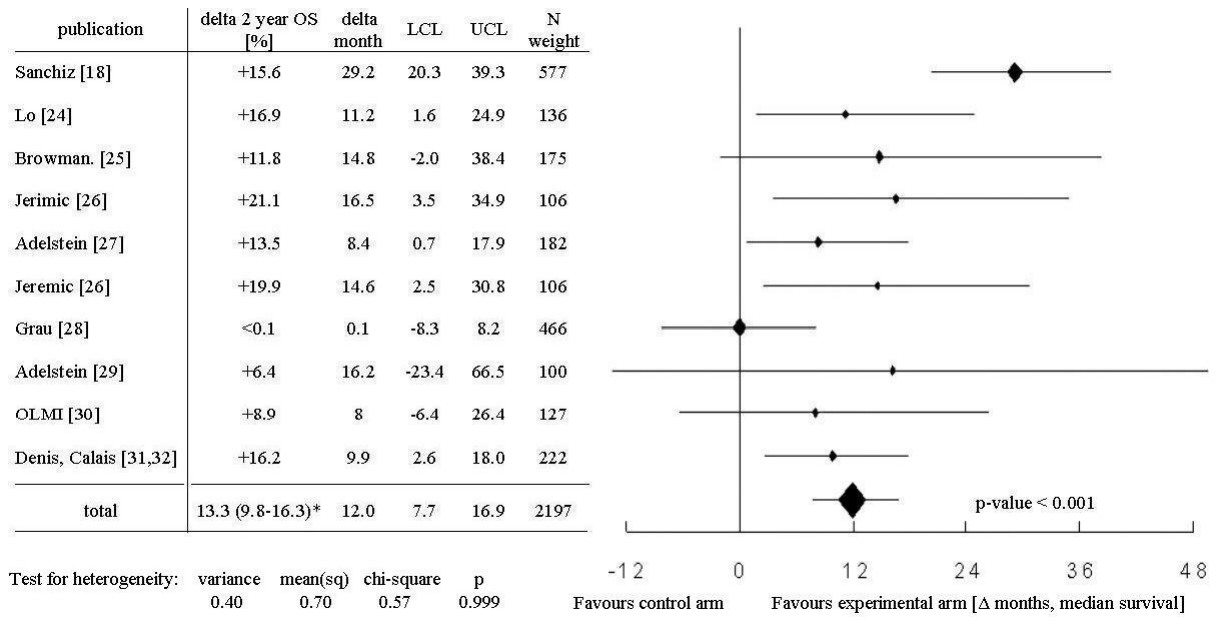
Difference of survival comparing conventionally fractionated radiotherapy with conventionally fractionated radiotherapy plus simultaneous chemotherapy using cisplatin, carboplatin, mitomycin C, and 5-fluorouracil as single drug or combinations of 5-FU with a platin-derivate (Group 1a). OS = Overall survival; * = 95% confidence limits of 2 year overall survival; delta months = differences of median overall survival; LCL = lower confidence limit of median survival; UCL = upper confidence limit of median survival; N = number of patients.

**Figure 2:**
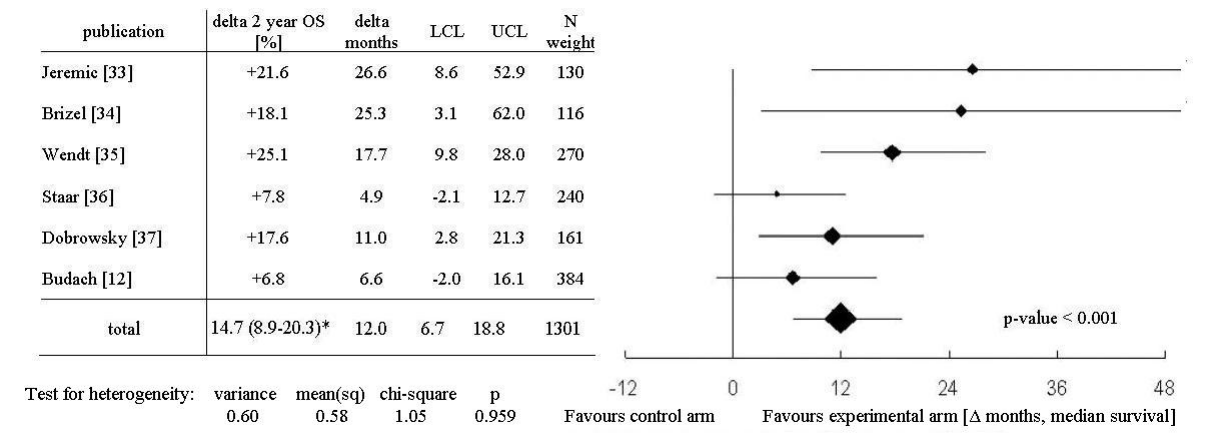
Difference of survival comparing hyperfractionated or accelerated radiotherapy with hyperfractionated or accelerated radiotherapy plus simultaneous chemotherapy using cisplatin, carboplatin, mitomycin C, and 5-FU as single drug or combinations of 5-FU with one of the other drugs (Group 1b). OS = Overall survival; * = 95% confidence limits of 2 year overall survival; delta months = differences of median overall survival; LCL = lower confidence limit of median survival; UCL = upper confidence limit of median survival; N = number of patients.

**Figure 3:**
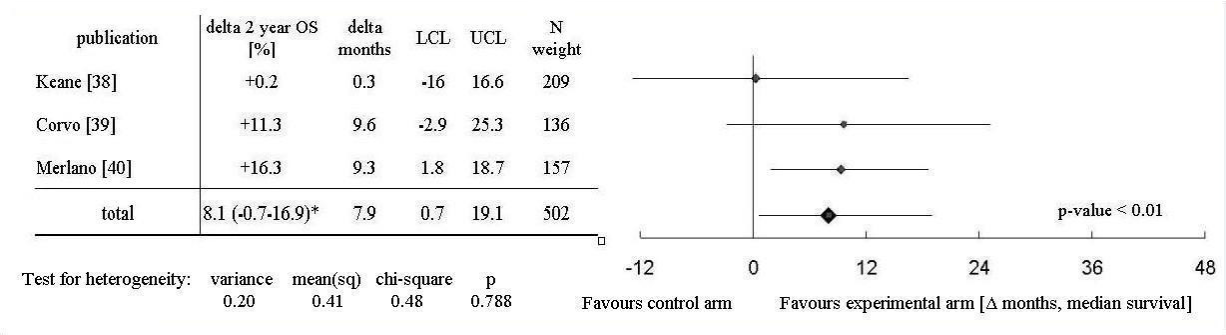
Difference of survival comparing altered fractionated radiotherapy with altered fractionated radiotherapy plus chemotherapy using combinations of 5-FU with cisplatin or mitomycin C (Group 1c). This group of trials showed a relevant difference/prolongation in overall treatment time (delta 7 – 28 days) between standard and experimental treatment arm, caused by split course treatment or rapidly alternating radio-chemotherapy. OS = Overall survival; * = 95% confidence limits of 2 year overall survival; delta months = differences of median overall survival; LCL = lower confidence limit of median survival; UCL = upper confidence limit of median survival; N = number of patients.

The analysis on CFRT in combination with simultaneous chemotherapy (CF-RT, group 1a) resulted in an overall survival benefit of 12.0 months (95% confidence limits (CI) 7.7 to 16.9 months) compared with CF-RT alone (p < 0.0001, figure 1, table 1). HFRT and/or AFRT in combination with simultaneous chemotherapy (HFRT/AFRT, group 1b) resulted in a similar overall survival benefit of 12.0 months (95% CI: 6.7 to 18.8 months) compared with HFRT/AFRT alone (p < 0.0001, figure 2, table 2). A smaller overall survival advantage of 7.9 months (95% CI: 0.7 to 19.1 months) was seen, if imbalanced radiation regimens were used in combination with simultaneous or alternating chemotherapy (Imbalanced-RT, group 1c) compared with radiotherapy alone (p < 0.001, figure 3, table 3). Differences in the gain of overall survival between group 1a, 1b, and 1c did not reach statistical significance.

**Figure 4:**
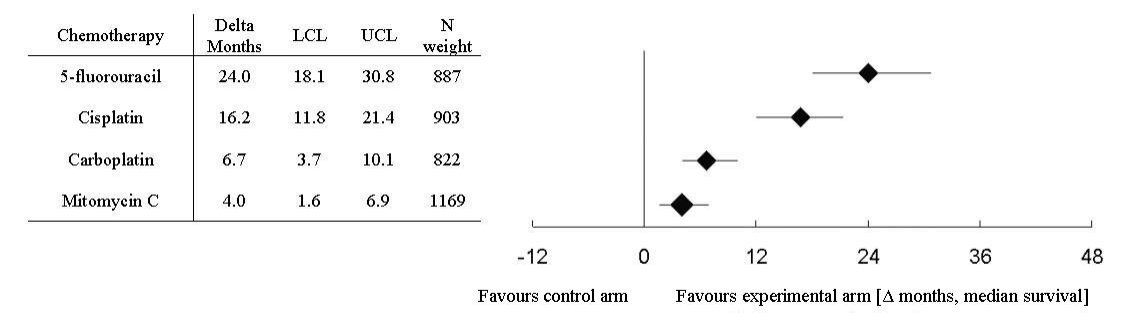
Difference of median survival with different drugs used for concomitant radiochemotherapy compared to radiotherapy alone, χ^2 ^= > p-value = 0.04. 5-fluorouracil: studies with simultaneus 5-fluorouracil chemotherapy [18,24,25,38]. Cisplatin: studies with simultaneus cisplatin based chemotherapy [26,27,29,33,34,35,39,40]. Carboplatin: studies with simultaneus carboplatin based chemotherapy [26,30,31,32,36]. Mitomycin C: studies with mitomycin C based chemotherapy [12,30,37]. LCL = 95% lower confidence limit, UCL = 95% upper confidence limit; N = number of patients.

The effect of different chemotherapeutic agents given simultaneously with radiotherapy, either conventionally fractionated or hyperfractionated/accelerated was analysed separately using the same studies: 5-fluorouracil as sole chemotherapy revealed an increase in overall survival time of 24.0 months (95% CI: 18.1 to 30.8 months, trial# 1, 2, 3), cisplatin based chemotherapy of 16.2 months (95% CI: 11.8 to 21.4 months, trial #4,5,8,12,13,14,15), carboplatin of 6.7 months (95% CI: 3.7 to 10.1 months, trial #6,9,10,16), and mitomycin of C 4.0 months (95% CI 1.6 to 6.9 months, trial #7,17,18), respectively (figure 4).

**Figure 5:**
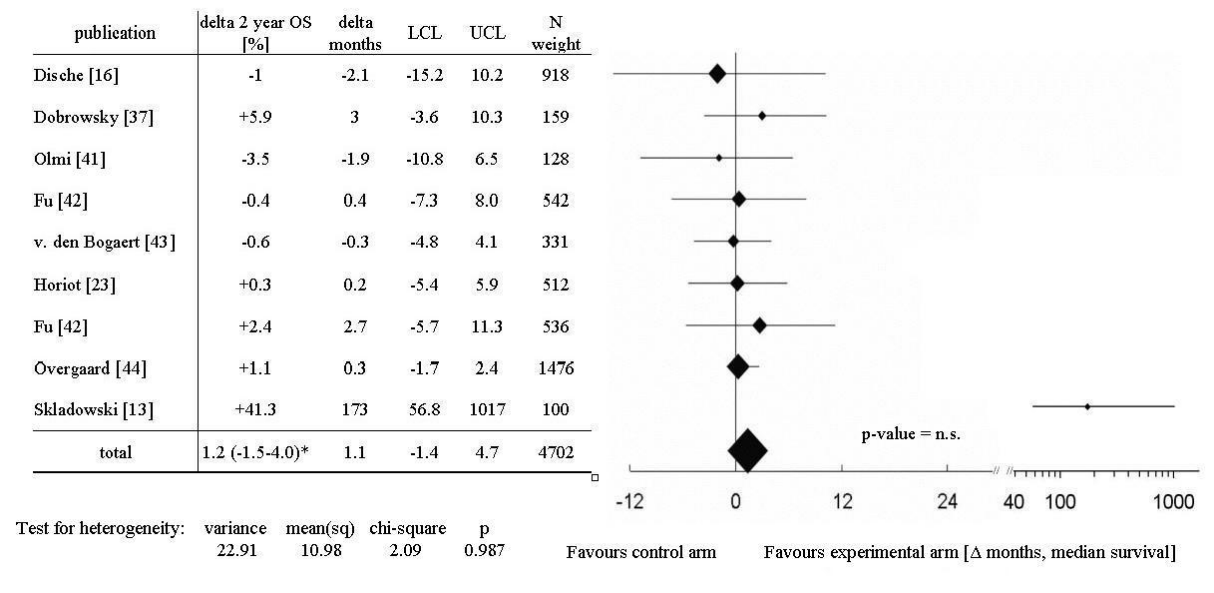
Comparison of accelerated radiotherapy with conventionally fractionated radiotherapy (Group 2). OS = Overall survival; * = 95% confidence limits of 2 year overall survival; delta months = differences of median overall survival; LCL = lower confidence limit of median survival; UCL = upper confidence limit of median survival; N = number of patients.

No survival benefit was observed in 8 out of 9 studies that tested AFRT versus CFRT (group 2). The large survival advantage reported from a smaller polish trial [13] that compared 5 days a week to 7 days a week radiotherapy is completely out of the range of the experience of all other studies. Nevertheless, including all studies no significant overall survival benefit (1.1 months, 95% CI -1.4 to 4.7 months) was observed comparing AFRT (group 2) with CFRT (figure 5, table 4).

**Figure 6:**
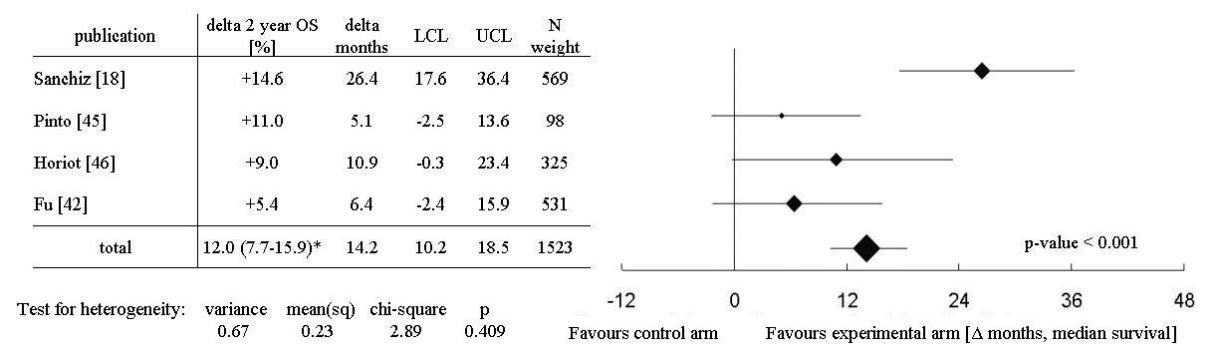
Comparison of hyperfractionated radiotherapy with conventionally fractionated radiotherapy (Group 3). OS = Overall survival; * = 95% confidence limits of 2 year overall survival; delta months = differences of median overall survival; LCL = lower confidence limit of median survival; UCL = upper confidence limit of median survival; N = number of patients.

Overall survival after HFRT (group 3) was consistently better than overall survival after CFRT in all four published trials. A survival benefit of 14.2 months (95% CI: 10.2 to 18.5 months) was observed in favour of HFRT (p < 0.001, figure 6, table 4).

The survival advantage of HFRT (figure 5) compared to CFRT was significantly larger than the modest (non significant) benefit of AFRT (figure 6) compared to CFRT radiotherapy (likelihood ratio test χ^2 ^= 36.2, p-value < 10^-4^)

The chi-square tests for heterogeneity of each comparison regarding the survival gain showed no significant heterogeneity.

## Discussion

### Chemoradiation

We identified 20 trials with a total of 4000 patients fulfilling the strict entry criteria for the comparison of radiation therapy alone with simultaneous or alternating chemoradiation. A large survival benefit of 12.0 months was observed in favour of simultaneous chemoradiation irrespective of whether conventionally fractionated (Figure 1), hyperfractionated or accelerated radiation schedules (Figure 2) were used. A smaller, but statistically still significant survival advantage of 7.9 months was seen in the three studies that used prolonged overall treatment times (≥1 week) in the chemoradiation arms due to alternating chemoradiation or planned treatment breaks (Figure 3). Looking at the potential survival gain by cytostatic drugs, 5-FU as single drug and cisplatin as single drug or in combination with 5-FU exhibited the largest benefit. Carboplatin +/- 5-FU and mitomycin C +/- 5-FU were less effective, although the survival gain was statistically still significant (Figure 4). The survival benefit of 12.0 months seen for simultaneous chemoradiation schedules in studies that did not significantly prolong overall treatment time in the chemoradiation arm corresponds to an absolute survival gain of 13%–15% at 2 years. This benefit is almost twice as large as the benefit that has been reported form the update of the MACH-NC meta-analysis for simultaneous chemoradiation [5,6]. The MACH-NC meta-analysis included 50 studies on simultaneous chemoradiation regardless of the employed chemotherapy regimen and radiation dose. The inclusion of studies using bleomycin or methotrexate containing chemoradiation regimens into the MACH-NC meta-analysis is the main reason for the considerably smaller survival advantage. Methotrexate and bleomycin have both been shown to considerably enhance acute mucosal toxicity that will most likely result via consequential late effects in an increased late toxicity [7,14,15]. These observations and the lack of an obvious survival benefit prompted all large study groups to abandon the use of bleomycin and methotrexate in combination with simultaneous radiation therapy in head and neck cancer. Therefore, at least to our minds, it does not make sense to include theses studies into a meta-analysis, if one wants to estimate the real benefit of modern chemoradiation schedules in head and neck cancer patients. Similar arguments apply for studies that used, according to our current knowledge, subcurative total radiation doses. These studies were included into the MACH-NC meta-analysis, but excluded from the meta-analysis presented here, because we were only interested in the effect of chemotherapy in the curative setting. Furthermore, we used a more detailed distinction of the employed radiation fractionation schedules in combination with chemotherapy compared to the MACH-NC meta-analysis. The advantage of the MACH-NC meta-analysis is, however, that individual patient's data were used rather than available data from published materials as were mainly the basis for the meta-analysis presented here. One may argue against the "picking" of studies according to quite strict inclusion criteria. Our inclusion criteria are justified by the concentration on chemotherapy schedules still in use. The unusual end point (median survival time) was chosen because the model fit with the lognormal distribution showed that the conventional hazard ratios are not meaningful because they decline with time. The usual analysis methods based on the logrank test and the Cox proportional hazard model are therefore not appropriate, because they assume constant hazard ratios.

Regardless of these considerations, it is evident that simultaneous chemoradiation in locally advanced head and neck cancer results in a large survival benefit, if modern chemotherapy regimens are administered simultaneously with conventionally fractionated or hyperfractionated – accelerated radiotherapy, and that this benefit is probably underestimated by the update of the MACH-NC meta-analysis. The less pronounced survival advantage observed in studies with prolonged overall treatment times (average ~17 days) for combined treatments indicate that tumour cell repopulation is still a problem, when chemoradiation is used. Assuming a repopulation rate accounting for 0.3 Gy per day [16] and a reported average steepness of the dose response curves for head and neck cancer (γ_50 _~1.5 according to Okunieff et al. [17]) one would expect approximately 11% loss in loco-regional tumour control for a prolongation of the overall treatment time of 17 days. This can roughly be expected to translate into a survival advantage of 7%, representing approximately the magnitude of the decrease that was estimated in the meta-analysis presented here (Figure 1).

The size of the survival benefit in favour of chemoradiation was almost identical regardless whether mono-chemotherapy or poly-chemotherapy was used (data not shown). Differentiated by cytostatic drugs 5-FU and cisplatin appear to be more effective than carboplatin and mitomycin C. This is in agreement with the observations of the update of the MACH-NC meta-analysis, but cannot be regarded as prove for the superiority of the one or the other chemotherapy regimen. No randomized data are available for newer cytostatic drugs like taxanes that have been shown to be effective in head and neck cancer.

### Hyperfractionated and acceleration of radiation therapy

Radiobiological research predicted that by using hyperfractionated radiotherapy, one should be able to increase total radiation dose without an enhancement of late toxicity, but with a higher effectiveness of radiotherapy against the tumour. As shown in Figure 6, in terms of efficacy at the tumour, this prediction is unequivocally supported by the outcome of the clinical trials. An overall survival benefit has been observed in all four trials suggesting an average survival gain of 14.1 months in the meta-analysis. Even after exclusion of the trial performed by Sanchiz et al. [18] that has been criticized for questionable quality [19], the remaining trials still show a statistically significant survival benefit. However, it is important to keep in mind that we included solely trials that used at least a 5% increase in total dose in the hyperfractionated arm without a significant change in overall treatment time. In studies testing hyperfractionation without dose escalation a consistent survival advantage could not be demonstrated [3,20], that is, hyperfractionation per se does not result in a survival benefit, but dose escalation combined with hyperfractionation does. Hyperfractionation is simply the tool to enable dose escalation without an increase of severe late toxicity. The toxicities reported from the randomized trials suggest that hyperfractionation with dose escalation increases acute mucosal toxicity, but does not seem to induce significantly more severe late effects compared with conventional fractionation. However, late toxicity was not systematically assessed and reported in all studies so that a moderate increase of late toxicity cannot be excluded. The large survival benefit shown for hyperfractionation in combination with dose escalation clearly prevails the uncertainties regarding the toxicity.

Another prediction from radiobiological research [21] and retrospective clinical data [22] was that one should get better results by decreasing the overall treatment time of radiation therapy. The results of the CHART head and neck trial that compared 66 Gy in 46 days to 54 Gy in 12 days, proved that tumour cell repopulation during radiation therapy is an important clinical problem, although no gain in loco-regional tumour control or overall survival could be demonstrated for the accelerated radiation schedule in this study [16]. The concept of acceleration has been tested in a considerable number of randomised clinical trials (Table 4). Although many radiation oncologists regard accelerated treatment as beneficial for head and neck cancer patients, in the meta-analysis presented here, we were unable to demonstrate any significant survival benefit for accelerated radiation therapy (Figure 5). Eight out of nine studies were negative and the only trial showing a significant survival advantage is the smallest of all trials. This discrepancy deserves a closer look to the studies. Acceleration of radiotherapy was realized in three different ways: 1) by decreasing the overall treatment by more than 60% requiring a decrease in total dose of approximately 20% [16,37], 2) by a moderate reduction of the overall treatment time without a relevant compromise in total dose using split course radiotherapy regimens [23,41-43], and 3) by a moderate reduction of the overall treatment time without a relevant compromise in total dose using 6 or 7 fractions per week [13,44] or a concomitant boost radiotherapy regimen [42] without split. The first strategy trades dose for time and results neither in a gain of local tumour control nor of overall survival. Using the second strategy (split course), a significant improvement of loco-regional tumour control was observed in one [23] out of four studies. However, the better loco-regional tumour control in the EORTC 22851 trial [23] did not translate into an improved overall survival, which is likely to be a consequence of the significantly higher rate of severe late toxicities in the accelerated arm of the study resulting in an increase in the non cancer related death rate. The third strategy seems to work better. All three studies reported a significantly improved local tumour control [13,42,44] and one study, albeit the smallest, found a significant survival benefit [13]. The lack of a survival benefit in the DAHANCA trial [44] might be explained by the observation that in contrast to local tumour control, regional tumour control was not improved by acceleration and that routine neck dissection for persistent neck disease was not done in this study. The third trial used a concomitant boost radiation regimen [42] and observed a non significant improvement of overall survival.

Taken together, it appears that accelerated treatments using split course radiation schedules or reduced total doses results in no gain of loco-regional tumour control or overall survival. Accelerated treatments employing continuous radiation schedules without compromise in total dose improve local tumour control. The improvement of local tumour control in this subgroup of studies translated into a small, but non-significant survival benefit in the current meta-analysis. More data on this subgroup are needed.

## Conclusion

Radiation therapy combined with simultaneous 5-FU, cisplatin, carboplatin, and mitomycin C as single drug or combinations of 5-FU with one of the other drugs results in a large survival advantage irrespective the employed radiation schedule. If radiation therapy is used as single modality, hyperfractionation leads to a significant improvement of overall survival. Accelerated radiation therapy alone, especially when given as split course radiation schedule or extremely accelerated treatments with decreased total dose, does not increase overall survival.

## Competing interests

The author(s) declare that they have no competing interests.

## Authors' contributions

WB had the idea, coordinated the work and wrote the paper

TH and CB prepared the data for statistical analysis

VB provided a part of the data and gave advice regarding subgroup analysis

KD did the statistical analysis

## Pre-publication history

The pre-publication history for this paper can be accessed here:



## Supplementary Material

Additional File 1 contents a list of excluded studies Click here for file

Additional File 2 contents the plots of all studies including the estimates of the goodness of fits Click here for file

## Figures and Tables

**Table 1:** Conventionally fractionated radiotherapy with or without simultaneous chemotherapy using cisplatin, carboplatin, mitomycin C, and 5-FU as single drug or combinations of 5-FU with platinum-derivate (group 1a)

		control arm		experimental arm								
publication	trial #	dose [Gy]	RT time [days]	dose [Gy]	RT time [days]	DDP	Carbo	5-FU	FA	MMC	stage 4 [%]	neck N3 [%]

Sanchiz [18]	1	60	42.0	60	42.0			3750				30
Lo [24]	2	66	56.0	66	56.0			5500			41	38
Browman [25]	3	66	45.5	66	45.5			7200			55	14
Jerimic [26]	4	70	50.8	70	50.8	222					86	16
Adelstein [27]	5	70	49.0	70	49.0	300					94	28
Jeremic [26]	6	70	50.8	70	50.8		925				86	19
Grau [28]	7	66	47.0	66	47.0					30	53	4
Adelstein [29]	8	70	49.0	70	49.0	160		8000			72	31
Olmi [30]	9	68	47.3	68	47.25		900	12000			75	7
Denis, Calais [31,32]	10	70	49.0	70	49.0		840	7200			68	13

DDP = cisplatin cumulative dose [mg/m^2^]; Carbo = carboplatin cumulative dose [mg/m^2^]; 5-FU = 5-fluorouracil cumulative dose [mg/m^2^]; FA = folinic acid; MMC = mitomycin C cumulative dose [mg/m^2^]

**Table 2:** Optimised radiotherapy (hyperfractionation or acceleration) with or without simultaneous chemotherapy using cisplatin, carboplatin, mitomycin C, and 5-FU as single drug or combinations of 5-FU with one of the other drugs (Group 1b, no significant difference in overall treatment time between treatment arms)

		control arm		experimental arm								
publication	trial #	dose [Gy]	RT time [days]	dose [Gy]	RT time [days]	DDP	Carbo	5-FU	FA	MMC	Stage 4 [%]	neck N3 [%]

Jeremic [33]	11	77	49	77	49	210					82	18
Brizel [34]	12	75	42	70	40	120		6000				13
Wendt [35]	13	70.2	56	70.2	56	180		4200	1350			14
Staar [36]	14	69.4	38	69.4	38		700	6000			96	10
Dobrowsky [37]	15	55.3	17	55.3	17					20	82	13
Budach [12]	16	77.6	42	70.4	42			3000		20	94.5	14

DDP = cisplatin cumulative dose [mg/m^2^]; Carbo = carboplatin cumulative dose [mg/m^2^]; 5-FU = 5-fluorouracil cumulative dose [mg/m^2^]; FA = folinic acid; MMC = mitomycin C cumulative dose [mg/m^2^]

**Table 3:** Altered fractionated radiotherapy with or without chemotherapy using combinations of 5-FU with cisplatin or mitomycin C; only trials with a relevant prolongation in overall treatment time, caused by planned split course treatment or rapidly alternating radiochemotherapy (Imbalanced-RT, group 1c)

		control arm		experimental arm								
publication	trial #	Dose [Gy]	RT time [days]	Dose [Gy]	RT time [days]	DDP	Carbo	5-FU	FA	MMC	Stage 4 [%]	N3 [%]

Keane [38] CF split	17	50	28	50	56			8000		20	47	17
Corvo [39] alter. CF	18	75	42	60	56	400		4000			82	12
Merlano [40] alter. CF	19	70	49	60	56	400		4000			75	31

DDP = cisplatin cumulative dose [mg/m^2^]; Carbo = carboplatin cumulative dose [mg/m^2^]; 5-FU = 5-fluorouracil cumulative dose [mg/m^2^]; FA = folinic acid; MMC = mitomycin C cumulative dose [mg/m^2^]

**Table 4:** Comparison of accelerated or hyperfractionated radiotherapy with conventionally fractionated radiotherapy (Group 2/3)

		control arm		experimental arm				
publication	trial #	dose [Gy]	RT time [days]	dose [Gy]	RT time [days]	fractionation	Stage 4 [%]	N3 [%]

**group 2**		conventional fract.		acceleration				
Dische [16]	20	66	45.5	54	12	CHART	72	6
Dobrowsky [37]	21	70	49.0	55.3	17	CHART		12
Olmi [41]	22	68	47.3	65.6	42.7	AF split	70	7
Fu [42]	23	70	49.0	67.2	43.4	AF split	68	13
v. den Bogaert [43]	24	72.5	56.0	69.6	44.8	AF split	65	47
Horiot [23]	25	70	51.7	72	35	AF split	0	0
Fu [42]	26	70	49.0	72	42	concomittant boost		
Overgaard [44]	27	66–68	39.0	66–68	46.0	weekend	25	
Skladowsky [13]	28	70.6	54	70.75	40	weekend	17	0
**group 3**		conventional fract.		hyperfractionation				
Sanchiz [18]	29	60	42.0	70.4	44.8	HFX		30
Pinto [45]	30	66	46.2	70.4	44.8	HFX	54	25
Horiot [46]	31	70	51.1	80.5	49	HFX		15
Fu [42]	32	70	49.0	81.6	47.6	HFX	68	13
