# Supplementary material for: A meta-analysis of hyperfractionated and accelerated radiotherapy and combined chemotherapy and radiotherapy regimens in unresected locally advanced squamous cell carcinoma of the head and neck
Source: BMC Cancer. 2006 Jan 31;6:28. doi: 10.1186/1471-2407-6-28 (PMC1379652; doi:10.1186/1471-2407-6-28)
Supplement: Additional File 1 — contents a list of excluded studies [file 1471-2407-6-28-S1.doc]

Appendix 1

Listing of the excluded phase III trials

| *Group 1* | *Literature* | *Reason of exclusion* |
| --- | --- | --- |
| 1.1 | Smid L, Lesnicar H, Zakotnik B, et al. Radiotherapy, combined with simultaneous chemotherapy with mitomycin C and bleomycin for inoperable head and neck cancer: preliminary report. Int J Radiat Oncol Biol Phys 1995; 32: 769–75 | 1.1 –1.9  Methotrexate and bleomycin have both been shown to considerably enhance acute mucosal toxicity that will most likely result via consequential late effects in an increased late toxicity [7,14,15]. These observations and the lack of an obvious survival benefit prompted all large study groups to abandon the use of bleomycin and methotrexate in combination with simultaneous radiation therapy in head and neck cancer. |
| 1.2 | Vermund H, Kaalhus O, Winther F, Tausjø J, Thorud E, Harang R. Bleomycin and radiation therapy in squamous cell carcinoma of the upper aero-digestive tract: a phase III clinical trial. Int J Radiat Oncol Biol Phys 1985: 11: 1877–86. |
| 1.3 | Parvinen LM, Parvinen M, Nordman E, Kortekangas AE. Combined bleomycin treatment and radiation therapy in squamous cell carcinoma of the head and neck region. Acta Radiol Oncol 1985; 24: 487–89. |
| 1.4 | Eschwege F, Sancho-Garnier H, Gerard JP, et al. Ten-year results of a randomized trial comparing radiotherapy and concomitant bleomycin to radiotherapy alone in epidermoid carcinomas of the oropharynx: experience of the European Organization for Research and Treatment of Cancer. *NCI Monograph* 1988; **6:** 275–78. |
| 1.5 | Fu KK, Phillips TL, Silverberg IJ, et al. Combined radiotherapy and chemotherapy with bleomycin and methotrexate for advanced inoperable head and neck cancer.: update of a Northern California Oncology Group randomized trial. J Clin Oncol 1987; 5: 1410–18. |
| 1.6 | Knowlton AH, Percarpio B, Bobrow S, Fischer JJ Methotrexate and radiation therapy in the treatment of advanced head and neck tumors. Radiology. 1975 Sep;116(3):709-12 |
| 1.7 | Gupta NK, Swindell R.Concomitant methotrexate and radiotherapy in advanced head and neck cancer: 15-year follow-up of a randomized clinical trial. Clin Oncol (R Coll Radiol). 2001;13(5):339-44. |
| 1.8 | Salvajoli JV, Morioka H, Trippe N, Kowalski LP. A randomized trial of neoadjuvant vs concomitant chemotherapy vs radiotherapy alone in the treatment of stage IV head and neck squamous cell carcinoma.Eur Arch Otorhinolaryngol 1991; 249: 211–15. |
| 1.9 | Shetty P, Mehta A, Shinde A, Mazumdar A, Mingorani C. Controlled study in squamous cell carcinoma of base of tongue using conventional radiation, radiation with single drug and radiation with multiple drug chemotherapy. Proc ASCO 1985; 4: 152. |
| 1.10 | Stefani S, Chung TS. Hydroxyurea and radiotherapy in head and neck long-term results of a double blind randomized prospective study. Int J Radiat Oncol Biol Phys 1980; 6: 1398 | 1.10 – 1.11 Only trials comparing radiotherapy alone with radio-chemotherapy using simultaneous or alternating cisplatin, carboplatin, mitomycin C, and 5-fluorouracil (5-FU) as single drug or combinations of 5-FU with a platinum-derivate were eligible. |
| 1.11 | Forastiere AA, Goepfert H, Maor M, Pajak TF, Weber R, Morrison W, Glisson B, Trotti A, Ridge JA, Chao C, Peters G, Lee DJ, Leaf A, Ensley J, Cooper J.Concurrent chemotherapy and radiotherapy for organ preservation in advanced laryngeal cancer. N Engl J Med. 2003 Nov 27;349(22):2091-8 | Results of surgically and non-surgically treated patients were not reported separately. |
| 1.12 | Haffty BG, Son YH, Papac R, Sasaki CT, Weissberg JB, Fischer D, Rockwell S, Sartorelli AC, Fischer JJ: Chemotherapy as an adjunct to radiation in the treatment of squamous cell carcinoma of the head and neck: results of the Yale Mitomycin Randomized Trials. J Clin Oncol. 1997,15:268-276. | Results of surgically and non-surgically treated patients were not reported separately. |
| 1.13 | G. Fountzilas, E. Ciuleanu, M. Theophanopoulou, A. Kalogera-Fountzila, E. Samantas, E. Athanassiou, J. Tzitzikas, T. Ciuleanu, N. Zamboglou, N. Ghilezan. A randomized study of concomitant radiotherapy with cisplatin or carboplatin versus radiotherapy alone in patients with locally advanced non-nasopharyngeal head and neck cancer. A Hellenic Cooperative Oncology Group (HeCOG) phase III study. Proc Am Soc Clin Oncol 22: page 495, 2003 (abstr 1991) | Trials with published overall survival data, for at least two time points or survival curves, were eligible. This trial was published in abstract form with 3-year survival data only. |
| 1.14 | Weissler MC, Melin S, Sailer SL, Qaqish BF, Rosenman JG, Pillsbury HC 3rd: Simultaneous chemoradiation in the treatment of advanced head and neck cancer. Arch Otolaryngol Head Neck Surg. 1992,118:806-810. | Only trials with at least 60 randomised patients were considered. |
| *Group 2* |  |  |
| 2.1 | Hliniak A, Gwiazdowska B, Szutkowski Z, Kraszewska E, Kukolowicz P, Jarzabski A, Sochacka B, Mazurkiewicz M, Paprota K, Oliskiewicz W, Zadrozna O, Milecki P, Kubiak M, Czopkiewicz L, Jagas M, Gozdz S, Wieczorek A, Woytowicz A, Cisowska B, Magdziarz H, Nowakowski S, Kosniewski W, Laskosz I, Serafin A, Gradon E. A multicentre randomized/controlled trial of a conventional versus modestly accelerated radiotherapy in the laryngeal cancer: influence of a 1 week shortening overall time. Radiother Oncol. 2002;62:1-10. | Results of surgically and non-surgically treated patients were not reported separately. |
|  | Poulsen MG, Denham JW, Peters LJ, Lamb DS, Spry NA, Hindley A, Krawitz H, Hamilton C, Keller J, Tripcony L, Walker Q: A randomised trial of accelerated and conventional radiotherapy for stage III and IV squamous carcinoma of the head and neck: a Trans-Tasman Radiation Oncology Group Study. Radiother Oncol. 2001,60:113-122. | Only disease specific survival, but not overall survival data has been reported. |
| *Group 3* |  |  |
| 3.1 | Marcial VA, Pajak TF, Chang C, Tupchong L, Stetz J. Hyperfractionated photon radiation therapy in the treatment of advanced squamous cell carcinoma of the oral cavity, pharynx, larynx, and sinuses, using radiation therapy as the only planned modality: (preliminary report) by the Radiation Therapy Oncology Group (RTOG). Int J Radiat Oncol Biol Phys. 1987;13:41-7. | The overall treatment time varied more than 10% between treatment arms and the total radiation dose was not increased by at least 5% in the hyperfractionated arm of the study. |
| 3.2 | Marks R, Witherspoon B, Davis L, Rominger J, Marcial V. Hyperfractionation where do we stand – a preliminary RTOG report. Int J Radiat Oncol Biol Phys. 1978;4:139-140 Suppl 2. | No conventionally fractionated radiotherapy alone in the control arm (1.5Gy twice a day) and the total radiation dose was not increased by at least 5% in the experimental arm of the study. |
| 3.3 | Datta NR, Choudhry AD, Gupta S. Twice a day versus once a day radiation therapy in head and neck cancer. (Abstr.). Int J Radiat Oncol Biol Phys. 1989;17(Suppl.1):132 | Only loco-regional control, but not overall survival data have been reported. |
| 3.4 | Cummings BJ, Keane TJ, Pintilie M, et al. A prospective randomized trial of hyperfractionated versus conventional once daily radiation for advanced squamous cell carcinomas of the larynx and pharynx. Radiother. Oncol. 1996;40(Suppl.1):S30 | Hyperfractionation was defined as twice daily radiation treatments with <1.25 Gy per fraction. Experimental arm used 2x 1.45Gy/d, total dose 58Gy in 4 weeks. This trial was published in abstract form with 5-year survival data only. |
