# Supplementary material for: A meta-analysis of hyperfractionated and accelerated radiotherapy and combined chemotherapy and radiotherapy regimens in unresected locally advanced squamous cell carcinoma of the head and neck
Source: BMC Cancer. 2006 Jan 31;6:28. doi: 10.1186/1471-2407-6-28 (PMC1379652; doi:10.1186/1471-2407-6-28)
Supplement: Additional File 2 — contents the plots of all studies including the estimates of the goodness of fits [file 1471-2407-6-28-S2.doc]

Observed and expected survival curves for all 32 studies included in the meta-analysis.

Red and green crosses are the observations: green for control and red for experimental treatment. The blue and orange lines are the expected survival probabilities based on the lognormal distribution. Orange refers to the control arm and blue refers to the experimental therapy. The identification of the 32 studies is given in the following table. 26 out of 32 studies show an acceptable fit according to the $\chi^{2}$ goodness of fit test. The results of this test are given in the table at the end of this appendix. The p-values are shown in the figures.

| Study # | Group | Reference |
| --- | --- | --- |
| 1 | 3a | Adelstein et al. [27] |
| 2 | 3a | Sanchiz et al. [18] |
| 3 | 3a | Jeremic et al. [26] Carbo |
| 4 | 3a | Jerimic et al. [26] DDP |
| 5 | 3a | Browman et al. [25] |
| 6 | 3a | Lo et al. [24] |
| 7 | 3a | Adelstein et al. [29] |
| 8 | 3c | Corvo et al. [39] |
| 9 | 3c | Merlano et al. [40] |
| 10 | 3a | Olmi et al. [30] |
| 11 | 3c | Keane et al. [38] |
| 12 | 3a | Calais et al. [31]. update Denis et al. [32] |
| 13 | 3b | Budach et al. [12] |
| 14 | 3b | Brizel et al. [34] |
| 15 | 3b | Staar et al. [36] |
| 16 | 3b | Jeremic et al. [33] |
| 17 | 3b | Wendt et al. [35] |
| 19 | 3b | Dobrowsky et al. [37] |
| 21 | Acceleration | Dische et al. [16] |
| 22 | Acceleration | Dobrowsky et al. [37] |
| 23 | Acceleration | Fu et al. [42] Split |
| 24 | Acceleration | Fu et al. [42] Concomittant boost |
| 25 | Acceleration | Horiot et al. [23] |
| 27 | Acceleration | Olmi et al. [41] |
| 29 | Acceleration | Skladowski et al. [13] |
| 30 | Acceleration | v. den Bogaert et al. [43] |
| 32 | Hyperfractionation | Fu et al. [42] |
| 33 | Hyperfractionation | Horiot et al. [46] |
| 34 | Hyperfractionation | Pinto et al. [45] |
| 35 | Hyperfractionation | Sanchiz et al. [18] |
| 36 | 3a | Grau et al. [28] |
| 37 | Acceleration | Overgaard et al. [44] |


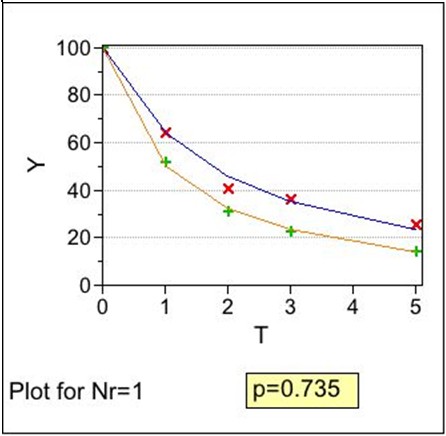

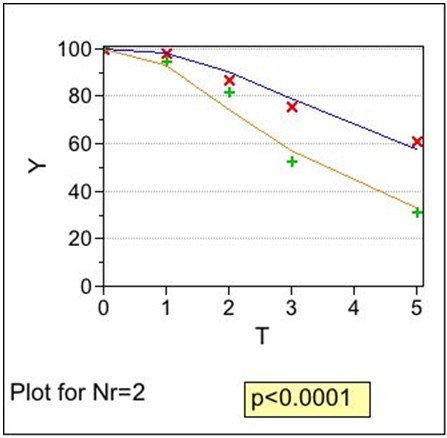

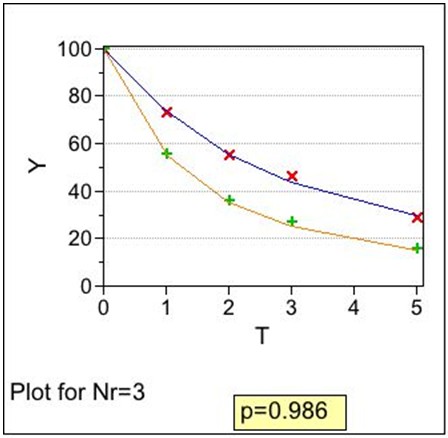

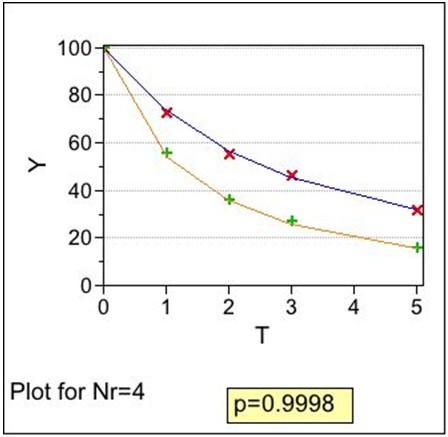

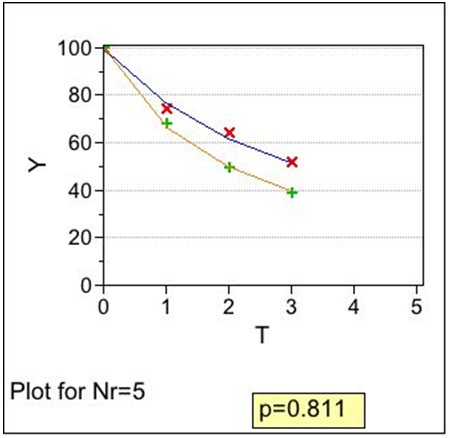

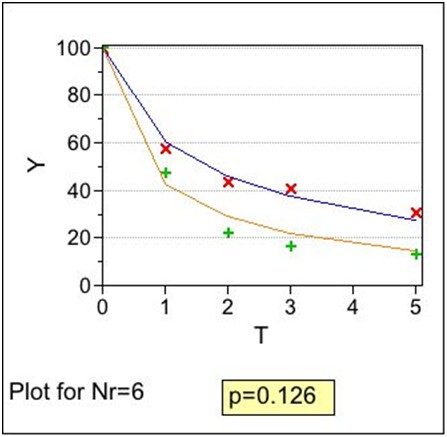

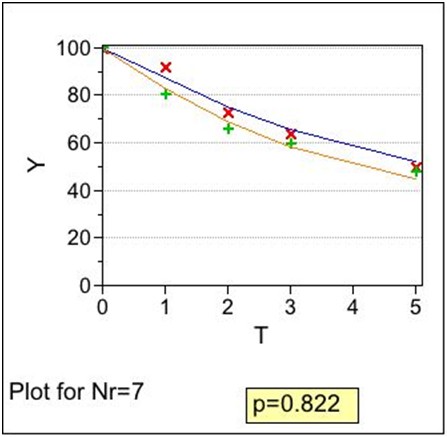

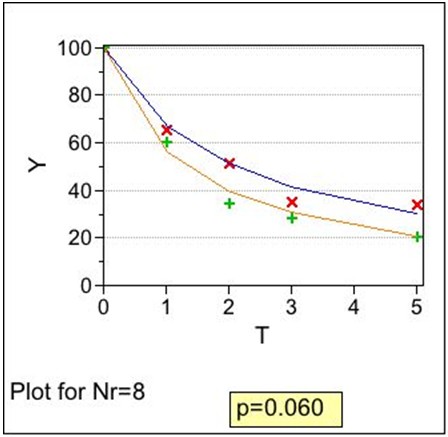

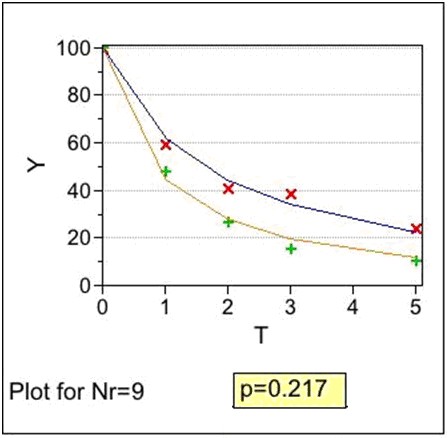

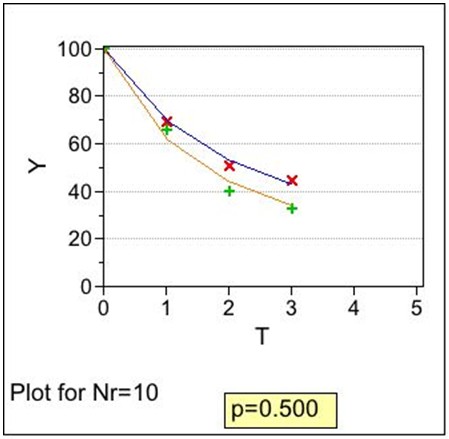

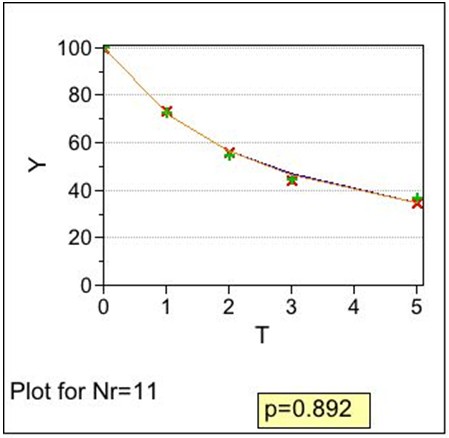

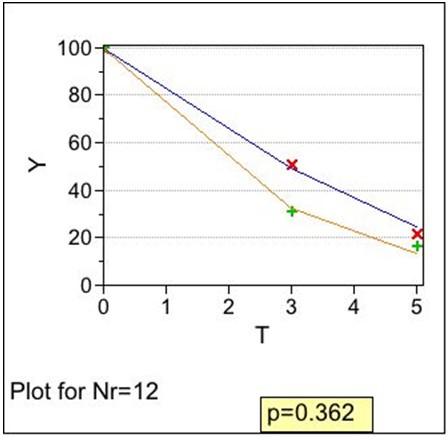

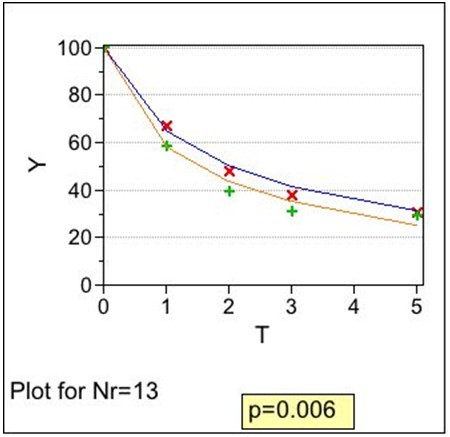

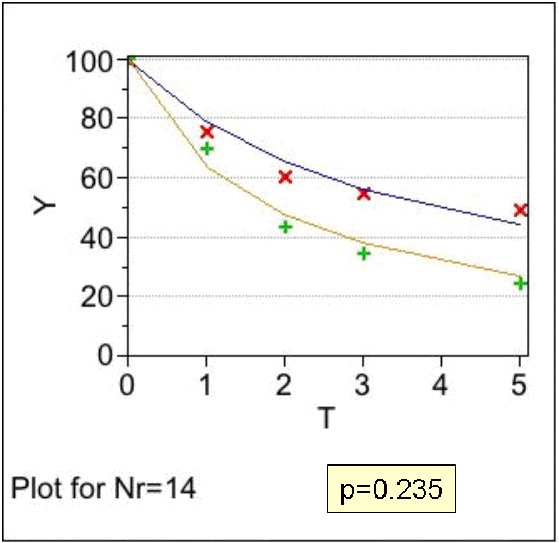

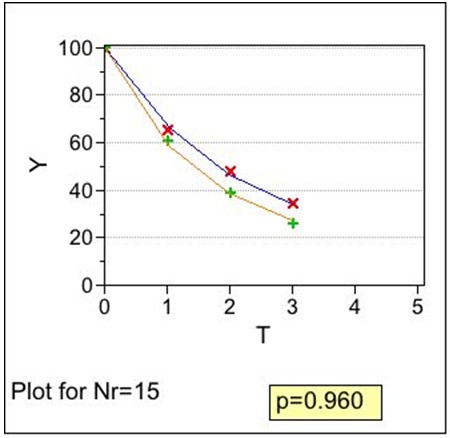

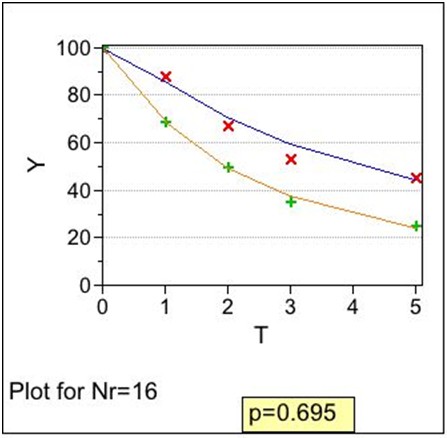

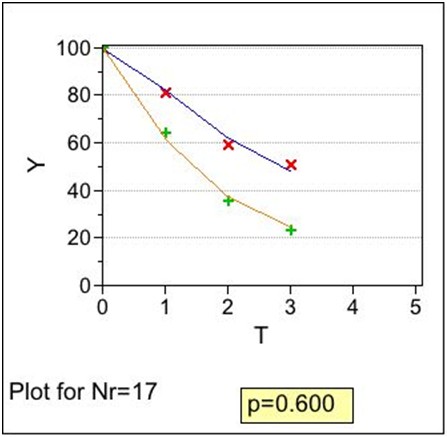

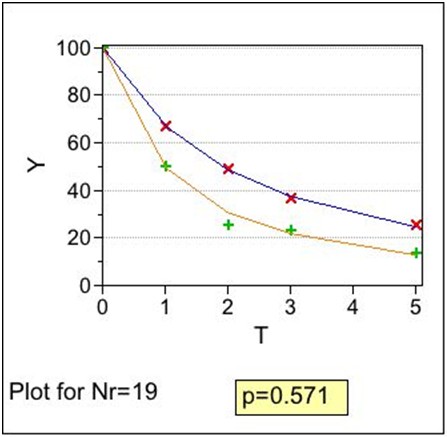

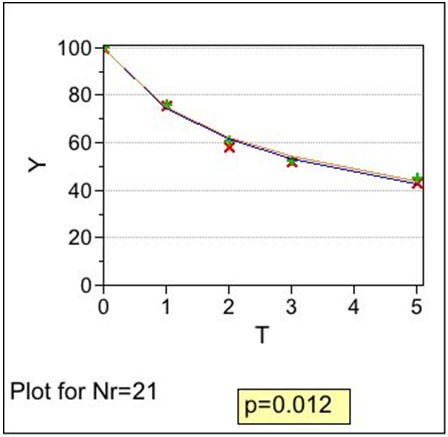

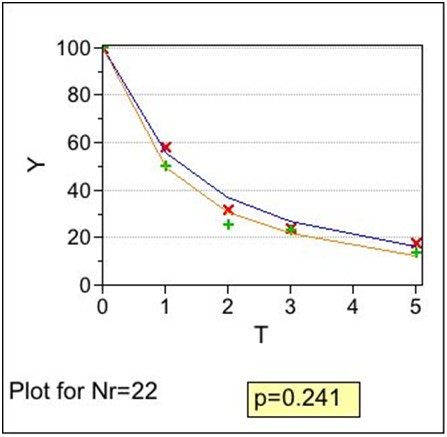

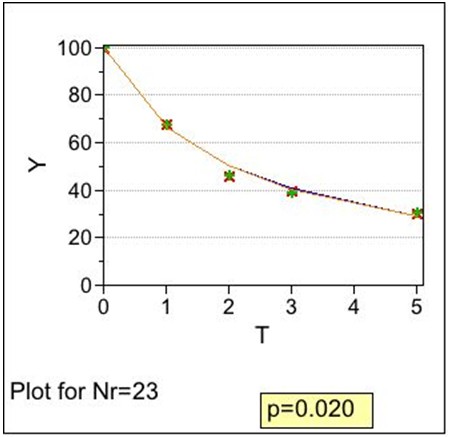

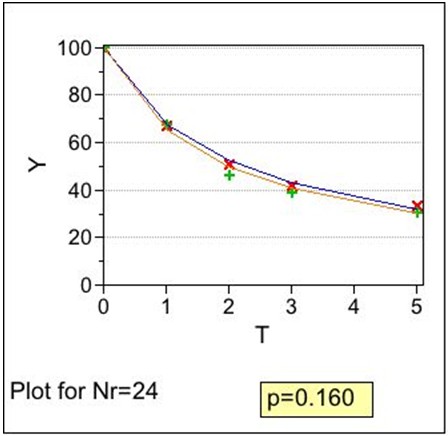

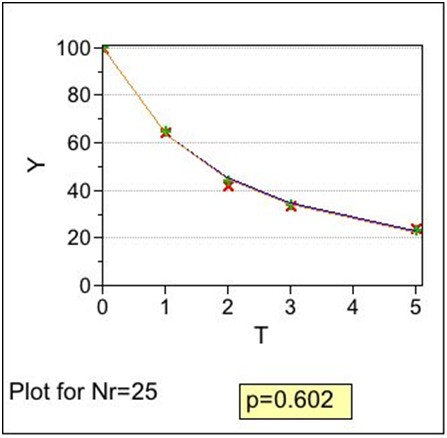

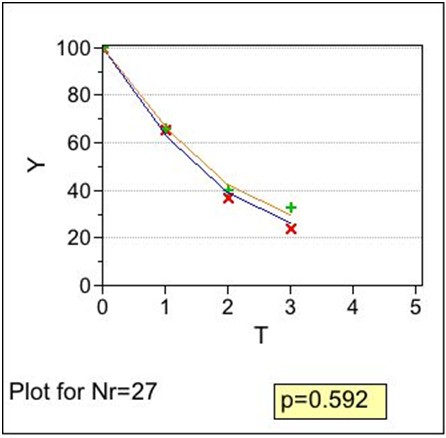

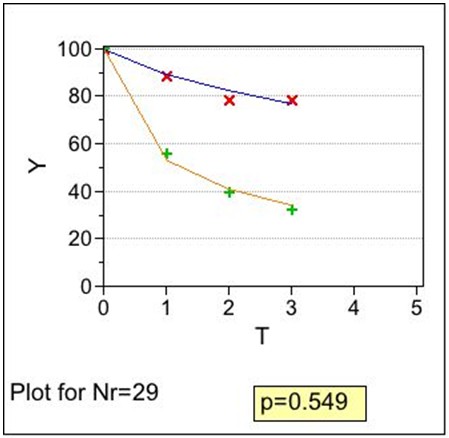

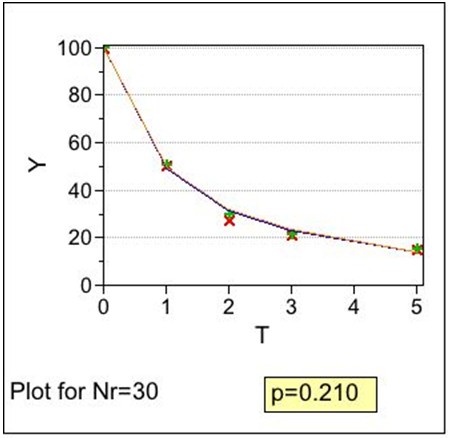

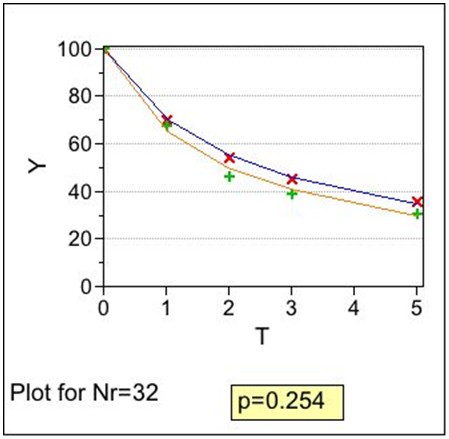

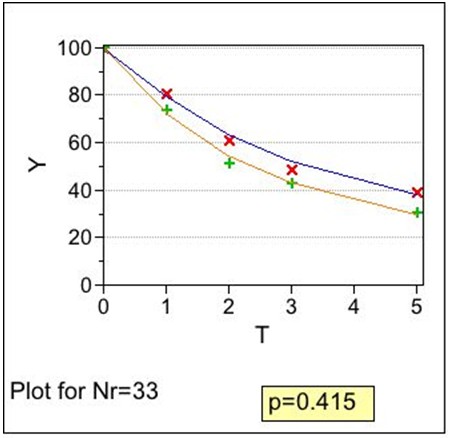

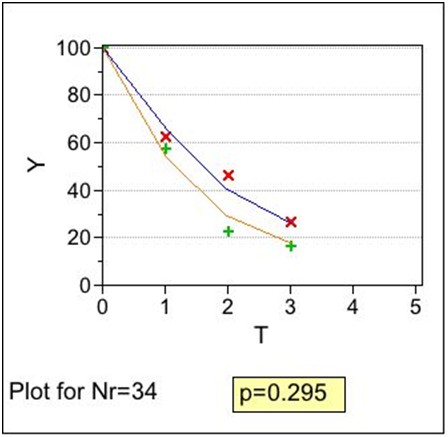

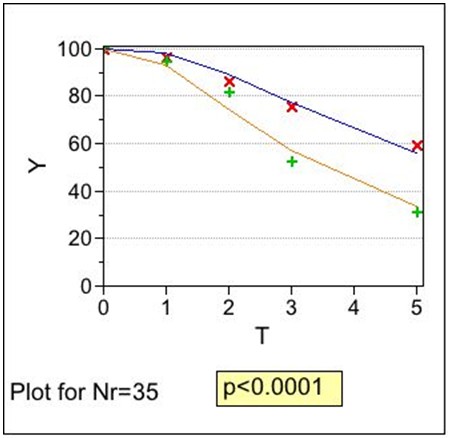

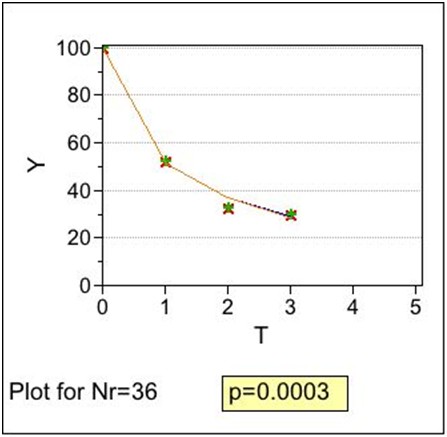

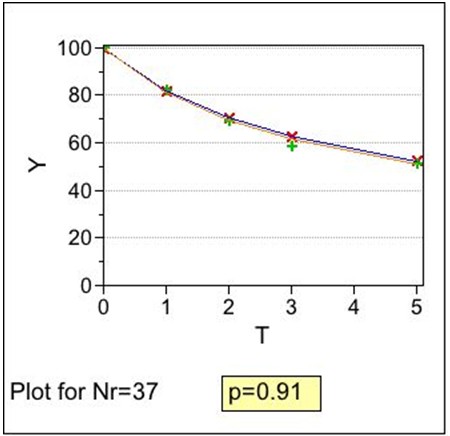

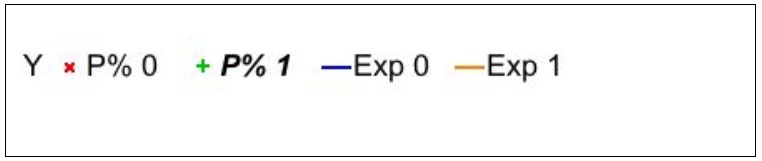


**Goodnees of fit**

| Nr | $\chi^{2}$ | p | suboptimal fit | n |
| --- | --- | --- | --- | --- |
| 1 | 4.38 | 0.735 | 0 | 182 |
| 2 | 37.7 | <0.0001 | 1 | 577 |
| 3 | 1.40 | 0.986 | 0 | 106 |
| 4 | 0.37 | 0.999 | 0 | 106 |
| 5 | 2.27 | 0.811 | 0 | 175 |
| 6 | 11.3 | 0.126 | 0 | 136 |
| 7 | 3.62 | 0.822 | 0 | 100 |
| 8 | 13.5 | 0.060 | 0 | 136 |
| 9 | 9.53 | 0.217 | 0 | 157 |
| 10 | 4.35 | 0.500 | 0 | 127 |
| 11 | 2.92 | 0.892 | 0 | 209 |
| 12 | 3.20 | 0.362 | 0 | 222 |
| 13 | 19.8 | 0.006 | 1 | 384 |
| 14 | 8.99 | 0.253 | 0 | 116 |
| 15 | 1.03 | 0.960 | 0 | 240 |
| 16 | 4.71 | 0.695 | 0 | 130 |
| 17 | 6.51 | 0.260 | 0 | 270 |
| 19 | 5.74 | 0.570 | 0 | 161 |
| 21 | 18.1 | 0.012 | 1 | 918 |
| 22 | 9.17 | 0.241 | 0 | 159 |
| 23 | 16.6 | 0.020 | 1 | 542 |
| 24 | 10.5 | 0.160 | 0 | 536 |
| 25 | 5.48 | 0.601 | 0 | 512 |
| 27 | 3.71 | 0.592 | 0 | 128 |
| 29 | 4.00 | 0.549 | 0 | 100 |
| 30 | 9.64 | 0.210 | 0 | 331 |
| 32 | 8.99 | 0.254 | 0 | 531 |
| 33 | 7.13 | 0.415 | 0 | 325 |
| 34 | 6.12 | 0.295 | 0 | 98 |
| 35 | 34.9 | <0.0001 | 1 | 569 |
| 36 | 23.0 | 0.0003 | 1 | 466 |
| 37 | 2.72 | 0.910 | 0 | 1475 |
